# Blind Watermarking of Color Medical Images Using Hadamard Transform and Fractional-Order Moments

**DOI:** 10.3390/s21237845

**Published:** 2021-11-25

**Authors:** Mostafa M. Abdel-Aziz, Khalid M. Hosny, Nabil A. Lashin, Mostafa M. Fouda

**Affiliations:** 1 Department of Information Technology, Faculty of Computers and Informatics, Zagazig University, Zagazig 44519, Egypt; eng_mostfa_it@yahoo.com (M.M.A.-A.); nlashin@zu.edu.eg (N.A.L.); 2 Department of Electrical and Computer Engineering, Idaho State University, Pocatello, ID 83209, USA; mfouda@isu.edu

**Keywords:** blind watermarking, color medical images, geometric attacks, fractional-order moments, fast Walsh–Hadamard transform

## Abstract

This paper proposes a new blind, color image watermarking method using fast Walsh–Hadamard transformation (FWHT) and multi-channel fractional Legendre–Fourier moments (MFrLFMs). The input host color image is first split into 4 × 4 non-interfering blocks, and the MFrLFMs are computed for each block, where proper MFrLFMs coefficients are selected and FWHT is applied on the selected coefficients. The scrambled binary watermark has been inserted in the quantized selected MFrLFMs coefficients. The proposed method is a blind extraction, as the original host image is not required to extract the watermark. The proposed method is evaluated over many visual imperceptibility terms such as peak signal-to-noise ratio (PSNR), normalized correlation (NC), and bit error rate. The robustness of the proposed method is tested over several geometrical attacks such as scaling, rotation, cropping, and translation with different parameter values. The most widely recognized image processing attacks are also considered, e.g., compressing and adding noise attacks. A set of combination attacks are also tested to analyze the robustness of the proposed scheme versus several attacks. The proposed model’s experimental and numerical results for invisibility and robustness were superior to the results of similar watermarking methods.

## 1. Introduction

Copyright protection over the Internet has become a big challenge because it deals with securing the transmitted digital content through the transition medium over the internet. Therefore, the use of digital image watermarking systems has become a hot research area. It is widely used in the copyright protection of images and security from several unwanted modifications of digital content, such as tampering and forgery over these transitions [1]. Digital watermarking can conceal any data type, including images, audio, video, and text in chosen digital media such as color images [2]. Two essential domains are used with the applied watermarking domain: the spatial working domain [3] and the frequency working domain [4]. In the spatial domain method, embedding the watermark can be realized by explicitly modifying the image pixels. It has the benefits of accessible execution at low computing costs. The most prevalent techniques in the spatial domain are LSB, correlation-based methods, histogram shifting, and speed spectral methods.

Furthermore, the most common techniques are primarily used in the frequency domain such as discrete Fourier transformation, discrete cosine transformation, discrete wavelet transformation, and singular value decomposition. Compared with spatial domain methods, the transform domain methods are more robust to several attacks, with high invisibility [5]. The watermark is concealed in the transformed coefficients in the transform domain, resulting in a minor change in image pixel correlation, preserving image quality much more [6].

According to the extraction method, there are three common types of watermarking systems: blind, non-blind, and semi-blind watermarking [5]. In a blind watermarking [7] system, the host cover image is not required in the watermark extraction process; it just needs the watermarked image. In non-blind [8] watermarking, the host and the watermarked images are both required to extract the watermark image. In semi-blind watermarking [9], the watermarked image and security key are required to engage the extraction process fully.

Concerning the desired security purpose of the watermarking models, there are three common characteristics: robustness [10], semi-fragile [11], and fragile [12] watermarking models. The robustness of the watermark system is that it resists a specific set of transformations such as geometric attacks. It is extensively used in copyright protection. The semi-fragile watermark system resists benign transformations only and can be detected after slight changes. It is used to detect and locate malignant transformations. The fragile watermark system cannot be detected after slight modification. It can be destroyed because it is highly sensitive to any slight modification; it is extensively used in tamper detection for proving integrity.

In recent years, many authors have introduced many watermarking methods in using color images; therefore, color images play a significant role in real-life applications such as copyright provisions and image authenticity for medical applications and other similar fields. 

Many researchers have presented realistic watermarking approaches in the spatial and frequency working domains. Parekh et al. [13] introduced a blind watermarking method in the spatial domain by modifying the luminosity component, Y, of the transformed YCbCr model. The host color image is split into 8 × 8 blocks, and a binary watermark has been embedded in a sorted correlation of values between corresponding blocks of sub-images. This scheme has been personalized, as it is low in robustness capabilities against various attacks. Su and Chen [14] introduced a watermarking scheme in the spatial domain to solve this problem. A binary logo image is embedded directly in distributed features in the direct current (DC) coefficients. This method proved to be a robust watermarking system against several geometric and standard signal attacks.

Owing to the robustness and indiscernibility of the watermarking systems in the frequency domain, many researchers proposed several watermarking strategies in this domain. Yuan et al. [15] proposed an image watermarking method for copyright protection. The color host image is divided into blocks, which are modified using (2D-DCT). The color logo image is embedded in the chosen intermediate coefficients of these selected blocks. The scheme is robust to most popular image processing attacks. Fares et al. [16] suggested a new image watermarking method based on discrete Fourier transform (DFT) of a fractional quaternion type. The watermark bits are concealed in the intermediate frequency band of the transformed RGB components by adjusting the least significant bits of the quantified magnitudes.

Many authors appealed to utilize the wavelet transformations in many watermarking schemes, in which it is a powerful tool that has been widely used in signal and image processing [17]. However, at the same time, it still has significant disadvantages such as (i) shift sensibility, any transitions in the input signal lead to unexpected changes in wavelet coefficients; (ii) inadequate directionality, since wavelet coefficients disclose only three spatial orientations; (iii) non-stationary signal, in view of the lake of phase information; (iv) computational complexity, especially for wavelet tree and numerous decomposition; and (v) complex accurate compilation of the mother wavelet and sub-bands mainly when applied in complicated fields. Our proposed method tried to overcome these significant problems using a hybrid technique by utilizing FWHT and MFrLFMs benefits to release a new, robust watermarking methodology.

Many researchers have touched upon the concept of perceiving the color image copyrights efficiently by using an advanced hybrid combination of the spatial and frequency domains techniques to achieve fast computation features of the spatial domain and at the same time take advantage of the durability of the frequency domain techniques. Yuan et al. [18] introduced a blind watermarking method based on a combination of spatial and frequency domains techniques. It was achieved by applying quantization steps on the DCT and discrete Hartley transformation (DHT) to embed a color logo image into the standard direct current frequencies blocks of the discrete cosine transform and DHT components. The watermark is extracted in a blind extraction manner by applying an inverse embedding process. The scheme is highly robust to many attacks. 

The remnant of this paper is structured as follows. Section 2 handles diverse related works. Section 3 includes the essential preliminaries that have been used in the proposed method. Section 4 illustrates the proposed watermarking methodology in a detailed manner. Section 5 documents the experiment’s numerical results with discussion, followed by the conclusion in Section 6. 

## 2. Related Work

Color medical images are the result of medical imaging of the interior of the human body for clinical analysis and medical intervention to reveal internal structures hidden by the skin and bones for diagnosing and treating disease. Medical images have also been established as a public database for medical purposes.

Due to the rapid increase in different types of attacks on color image watermarking systems, the successful extraction of the embedded watermark has become a common challenge in any current watermarking system because of higher sensitivity for different geometric, signal processing, and combination attacks. Any slight change in image pixel geometry leads to a false extraction process.

For this, many researchers have enhanced their efforts toward realizing many watermarking systems based on different invariant moments to attain the highest robustness and invisibility of watermarking systems. Ma et al. [19] introduced a robust watermarking method according to polar harmonic Fourier moments (PHFMs) and chaotic maps; accurate and robust PHFMs of the original cover image are computed and chosen depending on Gaussian numerical integration (GNI). The watermark was shuffled using a tent map and then concealed in the amplitudes of the selected PHFMs. The watermark extraction was done by applying PHFMs on watermarked images and the inverse of the embedding process in a blind extraction; this scheme is robust to different common geometric attacks. Yamni et al. [20] suggested a digital watermark algorithm based on fractional Charlier moments (FCMs) and singular value decomposition (SVD). In this method, the host cover images are split into 8 × 8 blocks. Then, the FCMs are computed for every block to obtain the FCM matrix, which SVD has decomposed by using adaptive pixel embedding with a quantization step. The method introduced the best superior fineness to reconstruct the watermarked images reasonably.

Wang et al. [21] introduced a watermarking algorithm depending on the TRHFMs’ computation of the input image. The selection of TRHFMs is randomly made using logistic maps to generate a zero-watermarked image by applying XOR between a binary watermark image and a binary feature image. This algorithm is robust to diverse types of attacks compared to similar methods. Ernawan [22] introduced a method using the Tchebichef-moments-based watermarking concept. The binary watermark is first shuffled using Arnold transformation and then concealed in the randomly selected Tchebichef moments matrix. This scheme is resistant to different classes of attacks, especially processing attacks. Singh et al. [23] introduced a watermarking system for medical images using PHFT, where the accurate computation of the chosen moment leads to a robust extraction process. Yamni et al. [24] introduced a blind watermarking method using radial Krawtchouk moments and an MLNCML chaotic system. The scheme was tested over standard signal processing and geometric attacks. Ernawan and Kabir [25] introduced a scheme using Tchebichef moments. The watermark logo is shuffled using an Arnold cat map and then embedded in the modified part of the selected moments over the chosen embedding blocks. Finally, the scheme is assessed for robustness versus widespread attacks.

Many researchers have revealed the use of quaternion-type moments (QTMs) in most current watermarking methods due to the ability and the advantages of dealing with cover images in a holistic manner [26]. It is more invariant to different image amendments such as rotation, translation, and scaling, since it can eliminate the transmutation parameters due to its invariance properties. It is also more efficient for computational analysis. As a result, Darwish et al. [27] introduced a scheme based on QLFMs and logistic maps. Logistic maps were employed on random selections of QLFM coefficients. The extraction process is the inverse operation of the embedding process. This method is successful in decreasing the computational time compared with other similar classical quaternion methods. This method achieves a high robustness level against more common attacks. 

Hosny and Darwish [28] proposed a watermarking method of the quaternion type using radial substituted Chebyshev moments (QRSCMs) in a holistic way to deal with color images. This method successfully overcame the limitations of other similar methods, such as low-level accuracy, low-speed computation, and instability changes over the coefficient matrix. Hosny and Darwish [29] introduced an image watermarking method based on fast and accurate QLFMs that first scrambled the binary watermark using Arnold transformation and then embedded this scrambled watermark in the quantized version of selected QLFMs using dither modulation. Li et al. [30] introduced a watermarking method on color images using Hadamard transformation and Zernike moments of a quaternion-type; quaternion Hadamard transform (QHT) is holistically mutated. Then the transformed components were divided into 8 × 8 non-overlapping blocks to apply Schur decomposition, with the watermark embedded on a selected block. The scheme proved its ability to overcome many counterattacks and its robustness against most common geometric attacks. In addition, the scheme lacks evaluation over combined attacks. Liu et al. [31] introduced a scheme on color images using QPHT block magnitudes and Bessel Key form distribution (BKF); the binary watermark was embedded in the amplitudes of the selected precise moment of the cover images blocks. This scheme introduced high imperceptibility compared with similar methods; however, it is still sensitive to most common geometric attacks. Xia et al. [32] introduced a method for copyrights provisions on medical images using QPHFMs. Computing QPHFMs generate the key image to the input CT images to reconstruct the features image, shuffling binary bit pixels using a chaotic system and then applying XOR between the generated feature image and the binary sequence image. Huynh-The et al. [33] proposed a blind color image watermarking scheme depending on optimal selective color channel bits embedding in two middle-frequency bands of quantized coefficients. Xia et al. [34] introduced a robust method on medical images based on QPHFMs using a sped-up robust feature (SURF) to generate multiple zero-watermarking systems. The method is superior in robustness to different complex attacks. Z. Xia et al. [35] introduced a Null-watermarking method on color medical images based on precise quaternion polar harmonic Fourier moments (PQPHFMs) and chaotic maps principles.

Similarly, Yamni et al. [36] introduced an image watermarking scheme based on FrCMMs and SVD for copyrights provisions. Liu et al. [37] introduced a fractional-order Krawtchouk watermark scheme depending on the decomposition of eigenvalues. This scheme is successful at achieving high robustness compared with other classical integer-order Krawtchouk moments. Yang et al. [38] derived a new fractional order of Jacobi–Fourier moments using mixed low-order moment attributes to increase image descriptor representation and robustness. Xiao et al. [39] derived FrDCMs by using the decomposition eigenvalues of the kernel matrix and applied it to a proposed watermarking scheme. Hosny et al. [40] proposed a new zero-watermarking method for color images based on accurate computation of multi-channel fractional Legendre-Fourier moments (MFrLFMs) and Arnold encryption to generate a robust watermarking system. Hosny et al. [41] proposed a new multi-channel fractional exponent moment order (MFrEM) to construct a robust color image watermarking method. In [42], the authors proposed a semi-blind watermarking method based on combined DWT-CT and Schur SVD decompositions; the method attained a high visual imperceptibility and robustness.

In the field of medical image authentication, many authors suggested many watermarking systems to achieve data privacy and at the same time preserve image quality as possible to manage disease diagnostics. Elbası [43] proposed a watermarking method on medical images based on M-SVD utilizing DWT to embed binary watermarks into medical images. The author succeeds in scoring accurate quality measurements of the watermarked images utilizing the proposed M-SVD technique. Huh and Kim [44] introduced a location-based mobile system that provides a healthcare structure for users to find patients’ location and disease diagnostic easily. Hosny and Darwish [45] proposed multiple zero-watermarking methods based on FrMGMs and scrambled to embed multiple watermarks into color medical images to present a high securing and robust watermarking system. Soualmi et al. [46] introduced multiple blind watermarking schemes based on LWT and QR decomposition for medical images authentication. Sun and Bo [47] proposed a blind watermarking model to embed a binary watermark image into a color medical image utilizing the benefits of wavelet PCA, low-frequency sub-bands, and HSV wavelet coefficients.

Based on this review of major revisions for most related methods, the proposed method’s contributions are as follows:

- Our method contributes to this evidence by using MFrLFMs. It is known for its stability and invariance to many geometric attacks, which keep robust capability over many attacks such as scaling, rotation, translation, etc.
- In addition, MFrLFMs are not restricted to integer-order values, which give them a high ability to represent the finest details in the image rather than their rival’s orthogonal polynomials of integer order.
- The use of Walsh–Hadamard transform comes under generalized Fourier transforms, known as low-computations compilations. Hadamard transform is a perpendicular function, which is composed of (−1 and +1) values only. Therefore, there is no data redundancy, which makes it widely used in many image processing analyses. FWHT is elastic to low-quality compression compared with other transformations such as traditional DCT and DWT, making it robust to most common image processing attacks.
- The proposed method introduced a new combination method of these multi-channel fractional Legendre–Fourier moments (MFrLFMs) and Hadamard transformation in a holistic way to achieve the main target of the proposed method, which represents robustness to both geometric and image processing attacks.
- The evaluation of our proposed method has been assessed for many common types of attacks and visual imperceptibility measurements.

## 3. Preliminaries

### 3.1. Fast Walsh–Hadamard Transformation Technique

The Hadamard transform is also known as the Walsh–Hadamard transform, Hadamard–Rademacher–Walsh transform, fast Walsh–Hadamard transform, or Walsh–Fourier transform and it comes under a summed-up class of Fourier transformation. It has the benefits of simple computational complexity $O\left(n^{2}\right)$ among all similar existing transforms [48]. It is orthogonal, symmetric, and has only two values (−1, +1), which results in isomorphism with no data redundancy. The FWHT has been extensively utilized in many image processing algorithms, digital steganography, and compression because it provides fast coefficient computation and fast reconstruction advantages. The fast Walsh–Hadamard transform is represented as follows:

(1) $$F(U,V)=\frac{1}{N}\times \;\left[H_{N}\right]\;\times \;f(x,y)$$

The F(U,V) refers to the FWHT of f(x,y), while the $\left[H_{N}\right]$ refers to the Hadamard matrix of squares.

The 4 × 4 Hadamard matrix is represented in the form

(2) $$\left[H4\right]=\left[\begin{array}{cc} H2 & \;\;\;\;H2\\ H2 & -H2 \end{array}\right]=\left[\begin{array}{c} 1\;\;1\;\;\;11\\ 1\;-1\;\;\;1\;\;\;-1\\ 1\;\;\;1\;\;-1\;\;-1\\ 1\;-1\;\;-1\;\;\;1\; \end{array}\right]$$

The main formula of the FWHT transformation is represented as

(3) $$H_{2^{P}}=\left[\begin{array}{cc} H_{2^{P}}-1 & H_{2^{P}}-1\\ H_{2^{P}}-1 & -H_{2^{P}}-1 \end{array}\right]\;for\;p=1,2,3,4,5,\ldots \ldots \ldots$$

where p values are based on the values of N, where $N=2^{P}$.

The inverse of the fast Walsh–Hadamard transform is represented as

(4) $$f(x,y)=\;\left[H_{N}\right]\;\times \;F(U,V)$$

where F(U,V) is the FWHT of the function, f(x,y).

### 3.2. MFrLFM Representation for RGB Color Image

Fractional-order polynomials are not restricted to integer-order values compared with other similar orthogonal moments, which give it a high ability to represent the fine details in an image rather than their rivals’ orthogonal polynomials of integer order [49]. These descriptors are also invariant to geometrical transformations (rotation, scaling, and translation). It is defined over a unit circle as follows:

(5) $$MFrM_{pq}\left(fc\right)=\frac{2p+1}{2\pi }\int_{0}^{2\pi }\int_{0}^{1}fc\left(r,\theta \right){\left[W_{pq}\left(r,\theta \right)\right]}^{*}rdrd\theta$$

where $\;W_{pq}$ represents the essential functions of the Legendre–Fourier moments of fractional orders.

The function fc(r,θ) denotes the RGB color images, the asterisk symbol “*” refers to the complex conjugate; p=|q|=0,1,2,3,4,5,6………∞.

The multi-channel fractional-order moments for all RGB color channels of the input image are computed using

$$f_{CR}\left(r,\;\theta \right),f_{CG}\;\left(r,\;\theta \right)\;\mathrm{and}\;f_{CG}\left(r,\;\theta \right)$$

Therefore, the original image is reconstructed by the reliance on the orthogonal property [50] of the fractional-order LFMs as follows:

(6) $$f_{C}^{recons}\left(r,\theta \right)=\overset{pmax}{\underset{p=0}{\sum }}\overset{qmax}{\underset{q=-qmax}{\sum }}MFrM_{pq}\left(f_{c}\right)W_{pq}(r,\theta )$$

where $f_{C}^{recons}\left(r,\theta \right)$ refer to the reconstructed image elements *R*, *G*, *B* of

$$f_{CR}^{recons}\left(r,\;\theta \right),\;f_{CG}^{recons}\left(r,\;\theta \right)\;\mathrm{and}\;f_{CB}^{recons}\left(r,\;\theta \right)$$

### 3.3. Geometrical Invariances of MFrLFMs

Robust watermarking algorithms are based on their invariance ability to RST geometric transformations. These RST invariants are derived and represented in a mathematical form in the following sub-sections.

#### 3.3.1. Rotational Invariance

Suppose that $f_{C}\left(r,\theta \right)$ denotes the original image and $f_{C}^{\beta }\left(r,\;\theta \right)$ denotes the rotated image with a rotational angle β where

(7) $$f_{C}^{\beta }\left(r,\;\theta \right)=f_{C}\left(r,\theta -\beta \right)$$

The MFrLFMs of $f_{C}^{\beta }\left(r,\;\theta \right)$ is computed as follows:

(8) $$MFrM{\;}_{pq}^{R}\left(f_{c}^{\beta }\right)=\frac{2p+1}{2\pi }\;\int_{0}^{2\pi }\int_{0}^{1}f_{c}\left(r,\theta \right)L_{p}(\alpha ,r)e^{-iq\theta \;\;}e^{-iq\beta \;\;}rdrd\theta \;=MFrM{\;}_{pq}\left(f_{c}\right)e^{-iq\beta }$$

Sparingly, it can be written as

(9) $$MFrM{\;}_{pq}^{R}\left(f_{c}^{\beta }\right)=e^{-iq\beta }MFrM{\;}_{pq}\left(f_{c}\right),\;C\;\in \left\{R,G,B\right\}$$

Since, $|e^{-iq\beta }|=1$; then

(10) $$\left|{MFrM\;}_{pq}^{R}\left(f_{c}^{\beta }\right)\left|=\right|{\;MFrM\;}_{pq}\left(f_{c}\right)\right|$$

Equation (10) proves the rotation invariance of MFrLFMs.

#### 3.3.2. Scale Invariance

Let $f_{C}\left(r,\theta \right)$ refer to the original image and $f_{C}^{S}\left(r,\;\theta \right)$ be the scaled image where

$$f_{C}^{S}\left(r,\;\theta \right)=f_{C}\left(\frac{r}{a}\;,\;\theta \right)$$

The MFrLFM of the scaled image $f_{C}^{S}\left(r,\;\theta \right)$ is computed as follows:

(11) $$MFrM{\;}_{pq}\left(f_{C}^{S}\right)=\frac{2p+1}{2\pi }\;\int_{0}^{2\pi }\int_{0}^{1}f_{c}^{S}\left(r,\theta \right)[W_{pq}(r,\theta )]*rdrd\theta \;=\frac{2p+1}{2\pi }\;\int_{0}^{2\pi }\int_{0}^{1}f_{C}\left(\overset{ˆ}{\underset{r}{\;}},\theta \right)L_{p}(\alpha ,a\hat{r})e^{-iq\theta \;\;}\hat{r}d\hat{r}d\theta \;\;\;=a^{2}\frac{2p+1}{2\pi }\;\int_{0}^{2\pi }\int_{0}^{1}f_{C}\left(\overset{ˆ}{\underset{r}{\;}},\theta \right)L_{p}(\alpha ,a\hat{r})e^{-iq\theta \;\;}\hat{r}d\hat{r}d\theta$$

The $L_{p}\left(\alpha ,a\hat{r}\right)\;$is the scaled fractional order.

Finally, the MFrLFM of the scaled image is computed as

(12) $$MFrM{\;}_{pq}\left(f_{C}^{S}\right)=\overset{p}{\underset{k=0}{\sum }}\frac{2p+1}{2k+1}\left(\overset{p}{\underset{i=k}{\sum }}a^{2i+2}C_{pi}d_{ik}MFrM_{kg}\left(f_{C}\right)\right)$$

#### 3.3.3. Translation Invariance

Translation invariance is realized when repositioning the original coordinates to synchronize with the centric points $\left(x_{c},y_{c}\right)$ of the color image [51] and $\left(x_{c},y_{c}\right)$ are defined as

(13) $$x_{c}=m_{10}\left(f_{R}\right)+m_{10}\left(f_{G}\right)+m_{10}\left(f_{B}\right))/m_{00}\;y_{c}=m_{01}\left(f_{R}\right)+m_{01}\left(f_{G}\right)+m_{01}\left(f_{B}\right))/m_{00}\;m_{00}=m_{00}\left(f_{R}\right)+m_{00}\left(f_{G}\right)+m_{00}\left(f_{B}\right)$$

The central MFrLFMs are represented mathematically as

(14) $$\frac{}{MFrM_{pq}}=\int_{0}^{2\pi }\int_{0}^{1}f_{C}\left(\bar{r},\bar{\theta }\right)[W_{pq}(\bar{r},\bar{\theta })]*\bar{r}d\bar{r}d\bar{\theta }\;=\int_{0}^{2\pi }\int_{0}^{1}f_{C}\left(\bar{r},\bar{\theta }\right)\;L_{pq}(\alpha ,\bar{r})]*e^{-iq\bar{\theta }}\bar{r}d\bar{r}d\bar{\theta }$$

### 3.4. Accurate MFrLFM Computation

**Figure 1:**
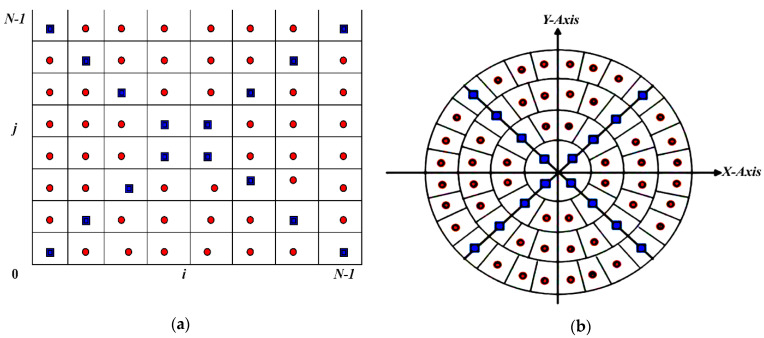
(**a**) Cartesian coordinates; (**b**) polar coordinates.

The selection of the accurate computation of MFrLFMs is one of the basic foundation rules of the proposed watermarking method. Any geometric distortion directly affects the robustness of the watermarking system over different geometric attacks, especially in medical images due to its high sensitivity to any distortion, which in turn leads to misdiagnosis. The computing of an accurate, kernel-based approach was introduced in [52]. MFrLFMs can be represented in polar coordinates (see Figure 1a) since they are symbolized in a unit circle. Each red point possesses eight point similarities, whereas each blue point possesses four similarities (see Figure 1b). The coefficient matrix of the MFrLFMs is calculated utilizing the equations:

(15) $$MFrM_{pq}=\frac{2p+1}{2\pi }\underset{i}{\sum }\underset{j}{\sum }k_{pq}\left(r_{i},\theta_{ij}\right)\;{\hat{f}}_{C}\left(r_{i},\theta_{ij}\right)$$

where

(16) $$k_{pq}\left(r_{i},\theta_{ij}\right)=I_{p}(r_{i})J_{q}(\theta_{ij})$$

The kernels functions [53] are defined as

(17) $$J_{q}\left(\theta_{ij}\right)=\int_{v_{ij}}^{v_{i,j+1}}e^{-\hat{i}q\theta }\;d\theta$$

(18) $$l_{p}\left(r_{i}\right)=\int_{U_{i}}^{U_{i+1}}L_{p\;}\left(\alpha ,r\right)\;rdr=\int_{U_{i}}^{U_{i+1}}R\left(r\right)dr$$

where

(19) $$R\left(r\right)=L_{p}\left(\alpha ,r\right)r$$

The upper and lower limits are

(20) $$V_{i,j+1}=\theta_{i,j}+\Delta \theta_{i,j}/2;\;V_{i,j}=\theta_{i,j}-\Delta \theta_{i,j}/2$$

(21) $$U_{i+1}=R_{i}+\Delta R_{i}/2;U_{i}=R_{i}-\Delta R_{i}/2\;$$

Compute $J_{q}\left(\theta_{i,j}\right)$ in the exact form

(22) $$J_{q}\left(\theta_{ij}\right)=\{\begin{array}{c} \frac{\hat{1}}{q}\left(e^{-\hat{i}\;q\;V_{i.j+1}}-e^{-\hat{i}\;q\;V_{i.j}}\right),q\neq 0\\ V_{i,j+1}-V_{i,j,\;\;\;,\;}q=0 \end{array}$$

Then, calculate the radial kernel $l_{p}\left(r_{i}\right)$ using suitable, accurate computation in [54] using the following equation:

(23) $$l_{p}\left(r_{i}\right)=\int_{Ui}^{Ui+1}R\left(r\right)dr\approx \;\frac{\left(U_{i+1}-U_{i}\right)}{2T}\;\overset{2T}{\underset{i=1}{\sum }}wR(U_{i}+\frac{\left(U_{i+1}-U_{i}\right)\left(l-0.5\right)}{2T}t)$$

Symbols “w” and “*t*” indicate the weights, where $T=2^{l}$ and l=0,1,2,……c−l, the value of t can be explained in respect of the integrity of $U_{i}$ and $U_{i+1}$ is the order of numeral integration, and the value of *w* is restricted where $\overset{c-1}{\underset{l=0}{\sum }}w=2$.

## 4. Proposed Blind Watermarking

In this paper, we propose a robust blind color image watermarking model. In the watermark extraction phase, the embedded binary watermark bits can be extracted from the selected MFrLFM coefficient magnitudes in a blind extraction manner. The scrambled watermark bit information is concealed in the accurate RST invariants’ selection of MFrLFM magnitudes of the host color image, transformed by fast Walsh–Hadamard transform to generate a robust watermarking system. The host image is not required; there is only a need for the color watermarked image to carry out the entire extraction procedures, preserving image quality as much as possible.

Blind watermark introduced a crucial function, especially in medical images. It is known for its ability to preserve image quality to guarantee an exact diagnosis of the disease and to protect patient data from unwanted malicious attacks. 

The idea behind deploying blind watermarking in our proposed method is to increase security, robustness, and imperceptibility. Furthermore, identity authentication must be provided for medical images, since every pixel in these images plays an essential role and represents essential patient data. Any distortion in these images’ pixels leads to disease misdiagnosis. It also has the advantage of a simple extraction; the watermark image can be extracted without needing the original host cover image, which reduces the time consumed during the extraction process and preserves image quality.

**Figure 2:**
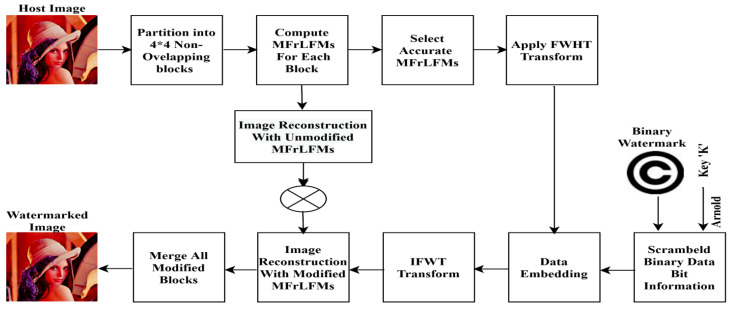
Proposed watermark embedding.

**Figure 3:**
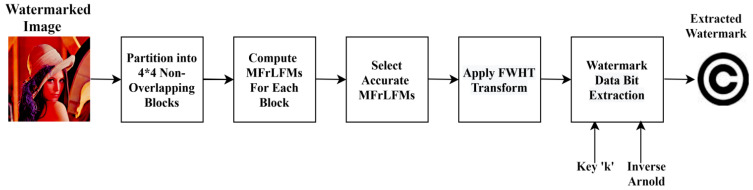
Proposed watermark extraction.

Sequentially, the detailed watermarking method for embedding and extraction processes is listed with illustrative diagrams as shown in Figure 2 and Figure 3, sequentially.

### 4.1. Watermarking Embedding Process

Assume that the input color host image is f, where

f={f(m,n),0≤m,n<N}

and the input watermark image is *W* of size *P* × *Q*, where

W={w(x,y)∈{0,1},0≤x<P,0≤y<Q}

which is to be concealed in the host color image. The embedding steps are discussed as follows:

Step 1: Scramble the binary watermark to banish the correlation between image pixels to add more reliability to the proposed method by using Arnold transformation [55] with arbitrarily chosen key (k), the watermark image is shuffled from *W* to *W*1, W1={W(x,y)∈{0,1},0≤x<P,0≤y<Q}. Transform it into a one-dimensional bit sequence as follows:

W2={W2(k)=w1 (x, y),0≤x<P,0≤y<Q ,k=x×Q+y,w2 (k)∈{0,1}}

Step 2: Split the input color image, *f*, into 4 × 4 non-interfering blocks, $B_{k}$,

(24) $$B_{k}=\left\{B_{k}\left(m,n\right),0\leq m,n<3\right\},K=1,2,\ldots \ldots N^{2}/16$$

Step 3: Compute MFrLFMs for each block, $B_{k}\left(K=1,2,\ldots \ldots N^{2}/16\right)$ using the maximum moment order *P* × *Q* as shown in (Section 3) in detail.

Step 4: Select robust and accurate MFrLFMs to carry the watermark; this is done by computing the magnitude values of MFrLFMs to ensure RST invariances. In the proposed method, we selected MFrLFMs with positive reiterations q≠4m,m∈Z to avoid redundancy as follows:

$$S=\left\{\;\left|FrM_{pq}\right|,\;q\neq 4m,m\in Z\right\}$$

By utilizing the key $k_{2},P\times Q$ coefficients of MFrLFMs, which denote the positions of the selected pixels in the image blocks, are selected randomly from the delicate set depending on the watermark bits to get the feature vector magnitude.

Step 5: Apply fast Walsh–Hadamard transform (FWHT) on selected MFrLFM coefficients using Equation (1).

Step 6: Embed the scrambled binary watermark, W1 bit information in the transformed MFrLFM magnitudes using dither modulation [56] using the following equation:

(25) $$m_{FrLFM^{\prime }}\left(M^{\prime }\right)=\left[\frac{m_{FrLFM}\left(x\right)-d_{x}\left(W_{1}\left(x\right)\right)}{\Delta }\right]*\Delta +d_{x}\left(W_{1}\left(x\right)\right),0\leq x<P\times Q\;d_{x}\left(1\right)=\frac{\Delta }{2}+d_{x}\left(0\right),d_{x}\left(0\right)\;\in \left[0,1\right]$$

The $m_{FrLFM^{\prime }}\left(M^{\prime }\right)$ denotes the modulated blocks. The mathematical symbols, [ .] , Δ and $d_{i}\left(.\right)$ refer to the rounding factor, quantization step, and dither modulation.

Step 7: Apply inverse fast Walsh–Hadamard transform (IFWHT) using Equation (4).

Step 8: Reconstruct the color watermarked image $f_{w}\left(r,\theta \right)$ as

(26) $$f_{w}\left(r,\theta \right)=f_{c}\left(r,\theta \right)-f_{c}^{M}\left(r,\theta \right)+f_{c}^{M\prime }\left(r,\theta \right)$$

where $f_{c}\left(r,\theta \right)$ is the host color image, $f_{c}^{M}\left(r,\theta \right)$ is the modified part, and $f_{c}^{M\prime }\left(r,\theta \right)$ is the unmodified part.

Step 9: Merge all 4 × 4 sub-blocks to get the color watermarked image finally $f_{w}\left(m,n\right).$

### 4.2. Blind Watermarking Extraction

The watermark extraction is done on a fully blind process. There is no need for the cover image; only the watermarked image is needed to generate an entirely successful extraction process by applying inverse operations of the embedding process. The extraction procedures are listed in the following steps in detail:

Step 1: Split the input watermarked image $f_{w}\left(m,n\right)$ into 4 × 4 non-interfering blocks, $B_{k}^{\prime }$,

(27) $$\;B_{k}^{\prime }=\left\{B_{k}^{\prime }\left(m,n\right),0\leq m,n<3\right\}\;\;,\;K=1,2,\ldots \ldots N^{2}/16$$

Step 2: Compute MFrLFMs for each block by using the method in Section 3.

Step 3: Select the MFrLFM coefficients’ feature vector,

$$M_{FrLFM}^{*}=\{m_{FrLFM}^{*}\left(i\right),0\leq i<p\times Q\}$$

As shown in the embedding process.

Step 4: Apply the fast Walsh–Hadamard transform (FWHT) on the selected MFrLFM magnitudes using Equation (1).

Step 5: Extract the watermark binary data bit information from the MFrLFM magnitudes by applying the inverse operation of Arnold transformation using the secret key, K, where the 1D binary bit series is extracted.

Step 6: Reconstruct the binary watermark image from the extracted one-dimensional binary bit sequence, $W_{1}^{*},$ by rearranging it into a two-dimensional binary image as

$$\;\;W^{\prime }=\{W^{*}\left(x,y\right),\in \left\{0,1\right\},0\leq x<p,0\leq y<Q\}$$

Step 7: Finally, obtain the binary extracted watermark image, $W^{\prime }$.

## 5. Experimental Results

**Figure 4:**
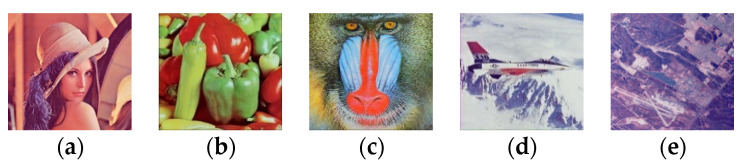

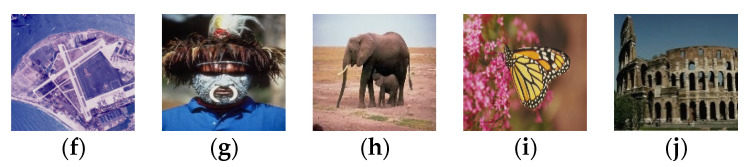
The host standard color images (**a**) Lena; (**b**) Peppers; (**c**) Baboon; (**d**) F16; (**e**) San Diego miramar; (**f**) San Diego north island; (**g**) Man; (**h**) Elephant; (**i**) Monarch; (**j**) Museum.

**Figure 5:**
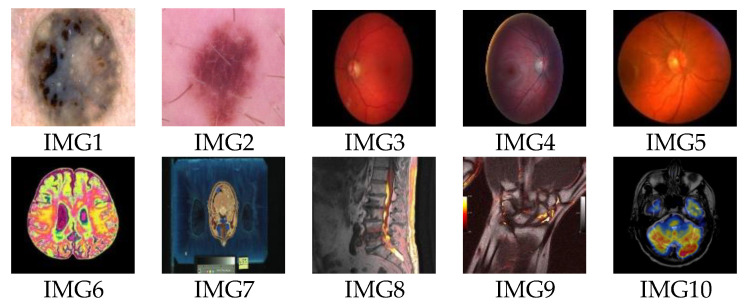
Selected host color medical images from datasets [57,58,59].

**Figure 6:**
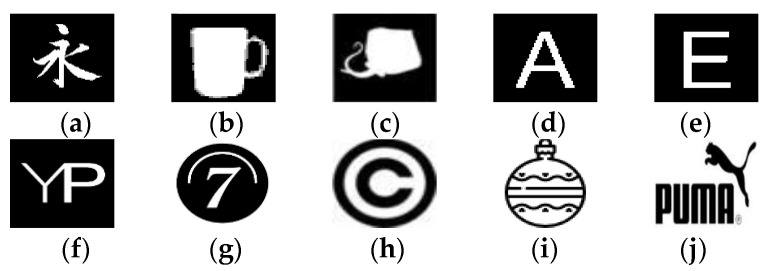
Selected binary watermark images (**a**–**j**).

In this part, various numeral simulations are enacted to assess the effectiveness of the proposed method according to the visual quality perception. PSNR and robustness evaluation has assessed these to several typical geometric, image filtering, and combination attacks conducted using NC and BER to measure the degree changes among pixels in the extracted binary watermark. The proposed method is tested over color host images of size 256 × 256 (shown in Figure 4) and color medical images selected from datasets [57,58,59] (shown in Figure 5). Binary watermark images are shown in Figure 6.

The attained numerical results are compared with other similar moment-based watermarking methods [28,29,41,60,61].

Additional comparisons with statistical watermarking methods [6,9,16,18,33] are also conducted to ensure the robustness and visual invisibility of the proposed methodology.

We document our experimental results according to the best and correct choice of α, where 0<α>3 and$\;\alpha \in R^{+}$.

Correspondingly, we tested different parameters values at α=0.7, 1.1, 2.3, and 2.6; from different experimental results, we noticed that the value of α at α=1.1 is the optimum choice, where there are no readjustments while using the optimum value Δ=0.2.

Experimental results documented using different image sizes (128 × 128 and 256 × 256) are depicted in the following sub-sections. The watermark logo images are of sizes 32 × 32 and 10 × 10.

### 5.1. Visual Imperceptibility

We utilized peak signal-to-noise ratio to measure and evaluate the proposed watermarking’s imperceptibility. Increasing the PSNR values leads to the high invisibility of the watermarking model. The PSNR is defined as

(28) $$PSNR\left(f,f_{w}\right)=10*{log}_{10}\left(\frac{MAX_{f}{}^{2}}{MSE}\right)$$

where f is the host color image and $f_{w}$ is the color watermarked image; MSE is the mean square error, and it is represented as

(29) $$Mse=\frac{1}{N^{2}}\overset{N}{\underset{i=1}{\sum }}\overset{N}{\underset{j=1}{\sum }}{\left[f_{w}\left(i,j\right)-f\left(i,j\right)\right]}^{2}$$

**Figure 7:**
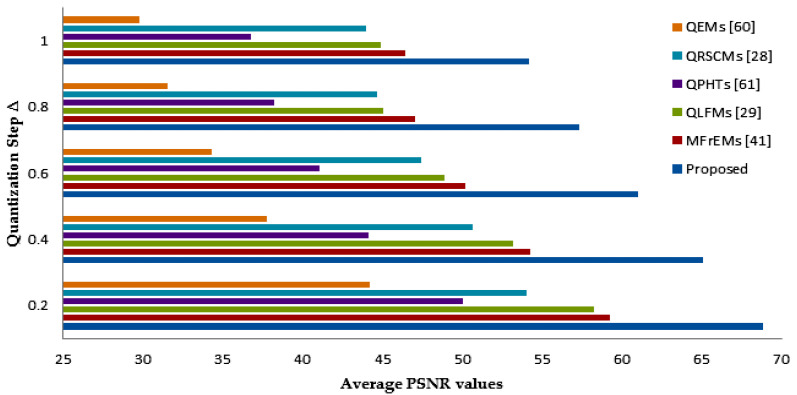
Average PSNR values using varying values of ∆.

The invisibility of the proposed methodology was evaluated at the value of α=1.1 and Δ values ranged from 0.2 to 1.0 in comparison with other existing moment-based methods [28,29,41,60,61], which in turn embedded binary watermarks of size 32 × 32 into the standard color images. The average PSNR values are indicated in Figure 7. As we notice, there is a reverse operation between the PSNR values, and PSNR values increased as Δ values decreased. The average PSNR values of the proposed watermarking method and other similar methods are shown in Table 1. From these values, we note the proposed method’s success in attaining high PSNR values up to ~68 dB compared with other similar methods, which ensures the high imperceptibility of our method compared to other similar methods.

**Figure 8:**
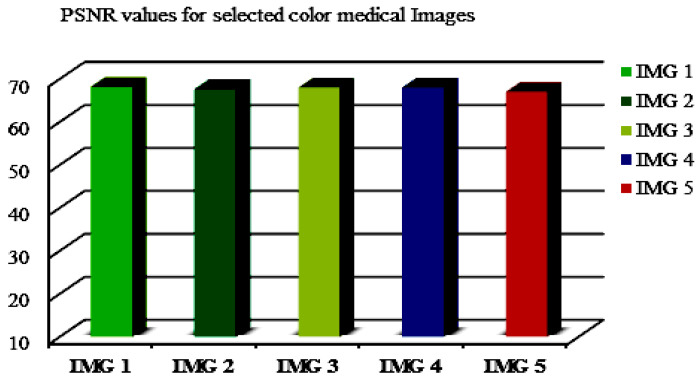
The PSNR values for selected color medical images at ∆ = 0.2.

The PSNR values for a chosen color medical image (named IMG 1–5) are also indicated in an illustrative example (Figure 8) with high PSNR values, ensuring that our proposed method is suitable for application on medical images in a reasonable manner.

### 5.2. Robustness Evaluation

The robustness of our method was evaluated over the bit error rate (*BER*) and normalized correlation (*NC*), which are formulated as

(30) $$BER=\frac{1}{P\times Q}\left(\overset{P}{\underset{x=1}{\sum }}\overset{Q}{\underset{y=1}{\sum }}{\left[W\left(x,y\right)-W^{*}\left(x,y\right)\right]}^{2}\right)$$

(31) $$NC=\frac{\sum_{x=1}^{P}\sum_{y=1}^{Q}\left[W\left(x,y\right)\times W^{*}\left(x,y\right)\right]\;\;}{\sum_{x=1}^{P}\sum_{y=1}^{Q}{\left[W\left(x,y\right)\right]}^{2}}$$

The robustness was evaluated over several categories of attacks, the first category comprised geometrics attacks such as rotation, scaling, cropping, magnification, and shearing attacks, with different parameter factors.

The second category included signal processing attacks such as jpeg, jpeg 2000, noising, salt and peppers, Gaussian, Gaussian low-pass, speckle noise attack, median filter attack, average filter attack, sharpen attack, histogram equalization, and motion blur attack. The third category included many common combination attacks.

The watermark logo was first resized to 10 × 10 and then embedded in the host color image by using and applying various geometric attacks on the watermarked image. Results for geometric attacks are shown in Table 2; several image processing attacks are also shown in Table 3, respectively.

The proposed method attained the highest robustness level against various geometrical and signal processing attacks.

**Figure 9:**
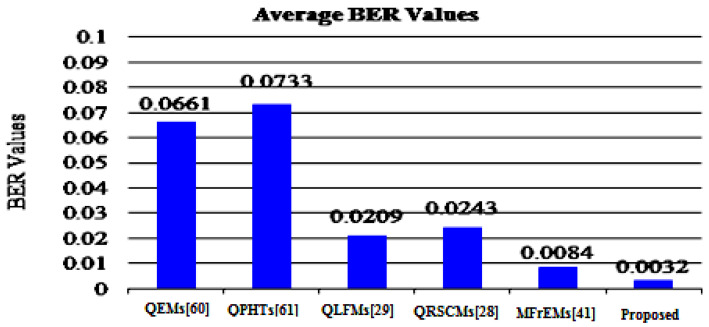
Average BER values of the proposed and compared methods for common attacks.

**Figure 10:**
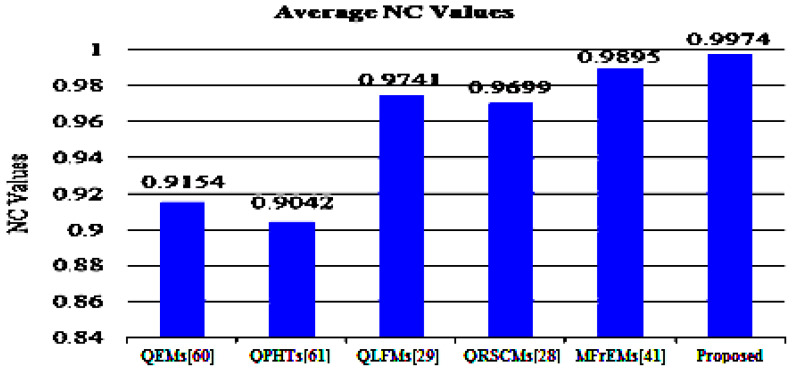
Average NC values of the proposed and compared methods for common attacks.

To assess the robustness of the proposed method and other mentioned methods, the average BER and NC values are represented in Figure 9 and Figure 10 for easy comparison. From these simulation results, (in the referred Figure 9 and Figure 10), we note that our method successfully scored the minimum average value (BER = 0.0032) among the compared methods. Moreover, our method scored the maximum average value of NC = 0.9974. From these compared values, we deduce that our method surpassed other existing methods.

The additional numerical experimental test was performed on a chosen binary watermark image called “cup” of size 32 × 32, which was embedded in the standard host image “Lena” and attacked using different common types of attacks, resulting in a fair comparison between the proposed method and moment-based methods [28,29,41,60,61]. The extracted watermark within the corresponding values of BER and NC is notated in Table 4.

**Figure 11:**
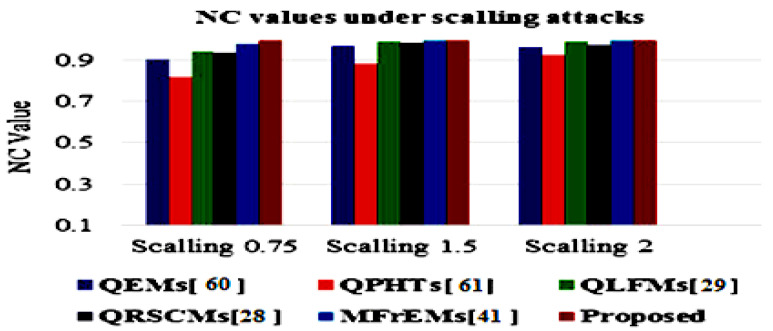
Graphical comparative of NC values under a scaling attack with different parameters.

**Figure 12:**
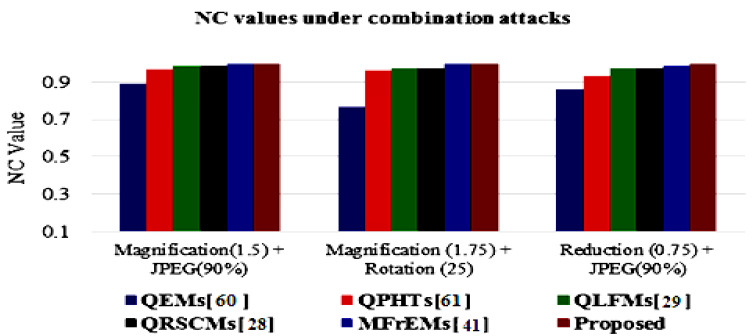
Graphical comparison of NC values using combination attacks with different parameters.

**Figure 13:**
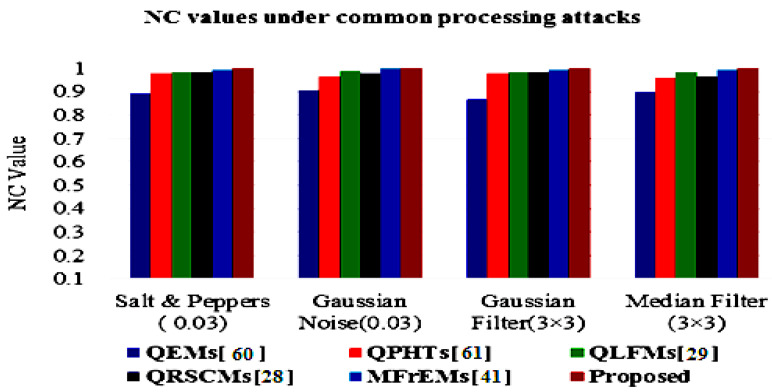
Graphical comparison of NC values under common noise and filtering attacks.

The simulation results of the proposed method and compared methods [28,29,41,60,61] of different NC values under various common attacks are also presented in a graphical simulation in Figure 11, Figure 12 and Figure 13. These results denote that the proposed method exceeded other existing methods and was robust to the various tested attacks.

From these results, we conclude that our method outperformed other existing methods. The watermark could be realized under various attacks suitably, and it seemed close to its original. In addition, the numerical values of BER and NC proved that the robustness of the proposed model surpassed other similar models.

We compared our method with more exiting statistical watermarking methods [6,9,16,18,33] in a comparison of maximum PSNR values of the watermarked images and the robustness of the extracted watermark over NC values under common attacks as shown in Table 5, from these values we can note that our proposed method succeeded in achieving a high robustness level and visual imperceptibility compared with others methods.

### 5.3. Capacity Evaluation

As there is a reverse operation between the payload capacity and visual image quality, increasing capacity degrades image quality (PSNR), so we considered this in our proposed method. The payload capacity of the proposed method was analyzed by the number of embedded bits per pixel (bpp). The maximum payload capacity for a chosen color host image of size 256 × 256 and watermark image of size 32 × 32 computed as (32 × 32)/(256 × 256 × 3) = 0.00520833 (bpp). It was obvious that our method scored satisfying results among the compared method as shown in Table 6, and at the same time preserved image quality as much as possible.

## 6. Conclusions

In this paper, a robust and efficient blind watermark method was proposed by utilizing FWHT and MFrLFMs on color images. The input binary watermark was scrambled using Arnold transformation to add more security and reliability to the proposed method. The accurate magnitude of MFrLFMs was selected to attain the goal of the watermarking system, which resulted in improved robustness and visual imperceptibility. The watermark bits were embedded in the modified magnitudes of the MFrLFMs based on the quantization step with optimum selected values. The proposed method successfully countered common geometric attacks, processing attacks, and combination complex attacks. The numerical results clearly show that our method surpassed existing similar methods with higher watermark invisibility, efficiency, and security, and it was suitable for medical images applications. In future work, we are looking to extend improvement to apply this method in a complete color image watermarking system to conceal color watermark logo images into color host cover images in a holistic way.

## Figures and Tables

**Table 1:** PSNR values using varying values of ∆.

Quantization Step ∆	QEMs [60]	QPHTs [61]	QRSCMs [28]	QLFMs [29]	MfrEMs [41]	Proposed Method
0.2	44.20	50.03	54.03	58.25	59.27	68.84
0.4	37.78	44.12	50.67	53.21	54.24	66.42
0.6	34.31	41.08	47.46	48.86	50.21	61.03
0.8	31.51	38.25	44.67	45.05	47.05	57.32
1.0	29.81	36.78	43.97	44.91	46.41	54.21

**Table 2:** Robustness results of the extracted watermark from the attacked image “Lena” using geometrics attacks, ∆ = 0.2.

Attacks		BER	NC	Extracted Watermark
No Attack		0	1	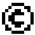
Rotation Attack	5°	0	1	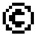
	15°	0	1	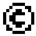
	25°	0	1	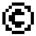
	65°	0	1	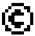
Scaling Attack	0.5	0.0023	0.9962	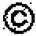
	0.75	0	1	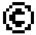
	1.25	0	1	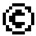
	1.5	0	1	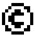
	2	0	1	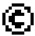
Translation Attack	(15,2)	0	1	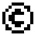
	(20,20)	0	1	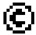
	(2,15)	0	1	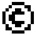
	(0,50)	0.0001	0.9998	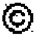
	(50,0)	0.0001	0.9998	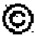
Shearing Attack	(0–1%)	0.0075	0.9932	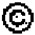
Magnification	(1.75)	0	1	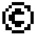
Cropping, Right	(25%)	0.0146	0.9820	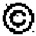
Cropping, Top	(25%)	0.0068	0.9916	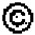
Cropping, Middle	(25%)	0	1	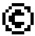

**Table 3:** Robustness results of the extracted watermark from the attacked image “Lena” using processing attacks ∆ = 0.2.

Attacks		BER	NC	Extracted Watermark
JPEG Compression	30	0	1	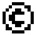
	40	0	1	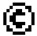
	50	0	1	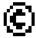
	60	0	1	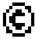
	70	0	1	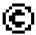
	90	0	1	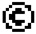
Motion Blur	(3,3)	0	1	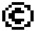
	(4,8)	0	1	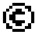
Lossy Compression (80)		0	1	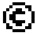
Salt and Pepper (0.03)		0	1	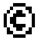
Gaussian (0.03)		0	1	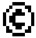
Gaussian Low-Pass (3,3)		0	1	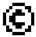
Poisson Noise		0	1	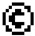
Speckle Noise Attack		0	1	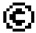
Median Filter Attack		0.0002	0.9972	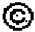
Average Filter Attack (3,3)		0.0003	0.9961	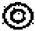
Sharpen Attack		0.0001	0.9998	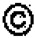
Histogram Equalization		0	1	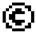

**Table 4:** Robustness evaluation for the proposed method and other exiting moment-based methods under common attacks.

Attacks		QEMs [60]		QPHTs [61]		QLFMs [29]		QRSCMs [28]		MFrEMs [41]		Proposed Method	
Rotation Angle	15°	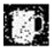	Ber = 0.0703 Nc = 0.9143	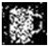	Ber = 0.1582 Nc = 0.7850	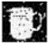	Ber = 0.0479 Nc = 0.9409	^ 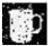 ^	Ber = 0.0479 Nc = 0.9408	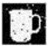	Ber = 0.0195 Nc = 0.9759	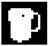	Ber = 0.0022 Nc = 0.9989
	35°	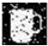	Ber = 0.0557 Nc = 0.9316	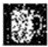	Ber = 0.1533 Nc = 0.7941	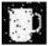	Ber = 0.0361 Nc = 0.9551	^ 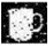 ^	Ber = 0.0449 Nc = 0.9445	^ 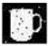 ^	Ber = 0.0176 Nc = 0.9782	^ 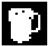 ^	Ber = 0.0050 Nc = 0.9953
	45°	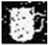	Ber = 0.0547 Nc = 0.9322	^ 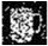 ^	Ber = 0.1514 Nc = 0.7945	^ 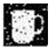 ^	Ber = 0.0459 Nc = 0.9439	^ 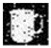 ^	Ber = 0.0459 Nc = 0.9434	^ 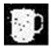 ^	Ber = 0.0195 Nc = 0.9758	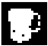	Ber = 0.0025 Nc = 0.9984
Scaling Factor	0.75	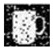	Ber = 0.0781 Nc = 0.9048	^ 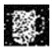 ^	Ber = 0.1377 Nc = 0.8146	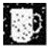	Ber = 0.0469 Nc = 0.9424	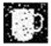	Ber = 0.0518 Nc = 0.9374	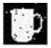	Ber = 0.0166 Nc = 0.9794	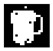	Ber = 0.0084 Nc = 0.9922
	1.5	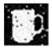	Ber = 0.0283 Nc = 0.9652	^ 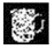 ^	Ber = 0.0898 Nc = 0.8825	^ 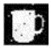 ^	Ber = 0.0098 Nc = 0.9880	^ 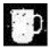 ^	Ber = 0.0127 Nc = 0.9843	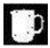	Ber = 0.0049 Nc = 0.9940	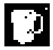	Ber = 0.0035 Nc = 0.9954
	2	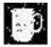	Ber = 0.0322 Nc = 0.9605	^ 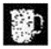 ^	Ber = 0.0586 Nc = 0.9251	^ 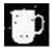 ^	Ber = 0.0078 Nc = 0.9904	^ 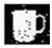 ^	Ber = 0.0205 Nc = 0.9745	^ 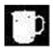 ^	Ber = 0.0039 Nc = 0.9952	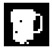	Ber = 0.0023 Nc = 0.9968
Translation	(H3,V3)	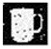	Ber = 0.0186 Nc = 0.9770	^ 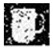 ^	Ber = 0.0625 Nc = 0.9237	^ 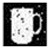 ^	Ber = 0.0205 Nc = 0.9745	^ 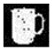 ^	Ber = 0.0107 Nc = 0.9867	^ 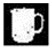 ^	Ber = 0.0039 Nc = 0.9952	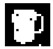	Ber = 0.0012 Nc = 0.9974
	(H6,V6)	^ 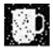 ^	Ber = 0.0479 Nc = 0.9412	^ 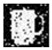 ^	Ber = 0.0635 Nc = 0.9226	^ 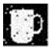 ^	Ber = 0.0215 Nc = 0.9736	^ 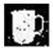 ^	Ber = 0.0205 Nc = 0.9747	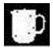	Ber = 0.0049 Nc = 0.9940	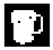	Ber = 0.0014 Nc = 0.9979
Compression	JPEG(80)	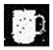	Ber = 0.0205 Nc = 0.9745	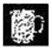	Ber = 0.0859 Nc = 0.8839	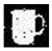	Ber = 0.0127 Nc = 0.9843	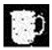	Ber = 0.0186 Nc = 0.9769	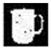	Ber = 0.0059 Nc = 0.9928	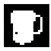	Ber = 0.0025 Nc = 0.9986
	JPEG(90)	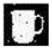	Ber = 0.0107 Nc = 0.9867	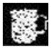	Ber = 0.0693 Nc = 0.9107	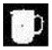	Ber = 0.0029 Nc = 0.9964	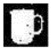	Ber = 0.0049 Nc = 0.9940	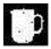	Ber = 0.0039 Nc = 0.9952	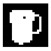	Ber = 0.0005 Nc = 0.9998
Magnification (1.5) + (JPEG, 90%)		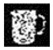	Ber = 0.0889 Nc = 0.8880	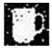	Ber = 0.0293 Nc = 0.9643	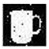	Ber = 0.0107 Nc = 0.9867	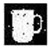	Ber = 0.0127 Nc = 0.9843	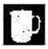	Ber = 0.0049 Nc = 0.9940	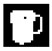	Ber = 0.0025 Nc = 0.9984
Magnification(1.75) + Rotation (25o)		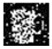	Ber = 0.1699 Nc = 0.7677	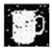	Ber = 0.0342 Nc = 0.9582	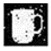	Ber = 0.0215 Nc = 0.9737	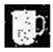	Ber = 0.0244 Nc = 0.9697	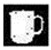	Ber = 0.0049 Nc = 0.9940	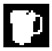	Ber = 0.0028 Nc = 0.9956
Reduction(0.75) + JPEG (90%)		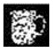	Ber = 0.1064 Nc = 0.8591	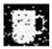	Ber = 0.0557 Nc = 0.9319	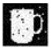	Ber = 0.0205 Nc = 0.9745	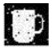	Ber = 0.0215 Nc = 0.9736	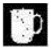	Ber = 0.0107 Nc = 0.9867	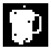	Ber = 0.0072 Nc = 0.9972
Noise Salt and Pepper (0.03)		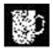	Ber = 0.084 Nc = 0.8907	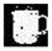	Ber = 0.0166 Nc = 0.9794	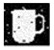	Ber = 0.0146 Nc = 0.9820	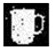	Ber = 0.0156 Nc = 0.9806	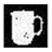	Ber = 0.0068 Nc = 0.9916	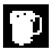	Ber = 0.0036 Nc = 0.9989
Gaussian Noise (0.03)		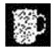	Ber = 0.0723 Nc = 0.9067	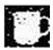	Ber = 0.0293 Nc = 0.9643	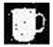	Ber = 0.0107 Nc = 0.9867	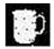	Ber = 0.0186 Nc = 0.9769	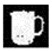	Ber = 0.0039 Nc = 0.9952	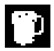	Ber = 0.0036 Nc = 0.9989
Gaussian Filter (3 × 3)		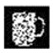	Ber = 0.1006 Nc = 0.8674	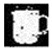	Ber = 0.0166 Nc = 0.9794	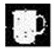	Ber = 0.0127 Nc = 0.9843	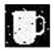	Ber = 0.0146 Nc = 0.9820	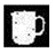	Ber = 0.0049 Nc = 0.9940	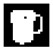	Ber = 0.0027 Nc = 0.9992
Median Filter (3 × 3)		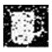	Ber = 0.0859 Nc = 0.8953	^ 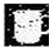 ^	Ber = 0.0342 Nc = 0.9582	^ 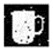 ^	Ber = 0.0137 Nc = 0.9831	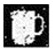	Ber = 0.0283 Nc = 0.9650	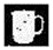	Ber = 0.0068 Nc = 0.9916	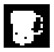	Ber = 0.0039 Nc = 0.9984

**Table 5:** Robustness evaluation of the proposed method and other exiting watermarking methods under common attacks.

Method	Prabha and Sam [6]	Rahman et al. [9]	Fares et al. [16]	Yuan et al. [18]	Huynh-The et al. [33]	Proposed Method
Max PSNR (dB)	64.3830	35.0406	42.42	44.4994	50.175	68.84
Rotation attack	0.8560	0.3567	0.9873	---	0.8534	0.9984
Scaling attack	0.9014	0.1928	0.96131	---	0.999	0.9968
JPEG 2000 attack	0.8577	0.9509	0.97639	0.9944	0.852	1
JPEG compression	---	0.9976	0.9998	0.9943	---	0.9998
Salt and peppers	0.9971	0.2569	0.8739	0.9849	0.996	0.9989
Gaussian	---	0.2186	0.94325	---	0.915	0.9989
Median filter	0.8370	0.9233	0.9011	0.9460	0.924	0.9984
Average filtering	---	0.8428	---	---	0.938	0.9961
Gaussian low-pass	0.9075	---	---	---	---	1
Gaussian filtering	---	0.9845	0.92237	---	0.997	0.9992
Blurring attack	0.9965		0.98814	---	0.965	1
Histogram Equalization	---	0.9449	0.96082	---	0.755	1
Zooming attack	---	0.9640	---	0.9995	---	1
Sharpen attack	0.9123	0.9179	0.98521	---	---	0.9998
Poisson noise	---	0.7168	---	---	---	1

**Table 6:** Capacity analysis for different methods.

Method	Watermark Image (Bits)	Cover Image (Pixels)	Bits/Pixels
Method [8]	32 × 32	512 × 512 × 3	0.00130208
Method [24]	64 × 64	512 × 512 × 3	0.00520833
Method [25]	64 × 64	512 × 512 × 3	0.00520833
QEMs [60]	16 × 16	256 × 256 × 3	0.00130208
QPHTs [61]	32 × 32	512 × 512 × 3	0.00130208
Proposed	32 × 32	256 × 256 × 3	0.00520833

## Data Availability

Not applicable.
